# A regional Canadian expert consensus on recommendations for restoring exercise and pulmonary function testing in low and moderate-to-high community prevalence coronavirus disease 2019 (COVID-19) settings

**DOI:** 10.1017/ice.2020.1339

**Published:** 2020-11-20

**Authors:** Sarah Khan, Kara K. Tsang, Dominik Mertz, Myrna Dolovich, Marcel Tunks, Catherine Demers, Kelly Hassall, Neil Maharaj, Karen Margallo, Maureen Cividino, Zain Chagla, MyLinh Duong

**Affiliations:** 1 Infection Prevention and Control, Hamilton Health Sciences, McMaster University, Hamilton, Ontario, Canada; 2 Department of Biochemistry and Biomedical Sciences, McMaster University, Hamilton, Ontario, Canada; 3 Faculty of Health Sciences, Aerosol Research, McMaster University, Hamilton, Ontario, Canada; 4 Division of Respirology, St Joseph’s Hospital, McMaster University, Hamilton, Ontario, Canada; 5 Division of Cardiology and Health Evidence and Impact, McMaster University, Hamilton, Ontario, Canada; 6 Respiratory Therapy Department St Joseph’s Healthcare Hamilton, Hamilton, Ontario, Canada; 7 Michael G DeGroote School of Medicine and Niagara Pulmonary Medicine & Diagnostics, McMaster University, Hamilton, Ontario, Canada; 8 Medical Diagnostic Unit, Hamilton Health Sciences, McMaster University, Hamilton, Ontario, Canada; 9 Occupational Health, St Joseph’s Hospital, McMaster University, Hamilton, Ontario, Canada; 10 Infection Prevention and Control, St Joseph’s Hospital, McMaster University, Hamilton, Ontario, Canada; 11 Division of Respirology, Hamilton Health Sciences, McMaster University, Hamilton, Ontario, Canada

*To the Editor—*Exercise and pulmonary function testing (PFT) are critical for the diagnosis, monitoring and management of cardiopulmonary disease. Previous respiratory pandemics have not impacted healthcare services to the same extent as coronavirus disease 2019 (COVID-19). Infection-specific guidance is now urgently needed to facilitate the resumption of pulmonary diagnostic services for low and moderate-to-high prevalence COVID-19 settings. Central to guidance development is the assessment of severe acute respiratory coronavirus virus 2 (SARS-CoV-2) transmission risk associated with these procedures, which are not recognized as aerosol-generating medical procedures (AGPs). However, they may carry risk due to the likelihood of generating coughs, exhaled respiratory droplets, and aerosols from the high ventilation and forced expiratory efforts. To date, there is no direct evidence for the risk of SARS-CoV-2 transmission in this context; thus, all guidelines are based on expert opinion from respiratory expert bodies.^1–6^ The aim of this document is to provide guidance for restoring exercise and pulmonary function testing in low and moderate-to-high prevalence settings of the COVID-19 pandemic. These recommendations are based on consensus from cardiopulmonary diagnostic service, occupational health, respiratory therapy, aerosol research, infection prevention and control (IPAC), and public health stakeholders to facilitate a uniform approach to the uptake and implementation of these recommendations. As further evidence emerges, revisions of these recommendations may be needed.

## Methods

We undertook a literature review of guidelines and studies published in English between January 1, 2012, and September 30, 2020, on aerosol and droplet generation including risks for viral transmission during exercise testing and PFT. We communicated with respirology community members across Canada to ascertain the scope of and variation in conducting these procedures during the pandemic. We met with regional experts and stakeholders to discuss the available evidence on prescreening, aerosol and droplet generation, transmission risk, personal protective equipment (PPE) appropriateness, and infection control measures relevant to SARS-CoV-2. We developed recommendations for the restoration of exercise testing and PFT in low and moderate-to-high prevalence COVID-19 settings, defined by the rate of community transmission (excluding institutional outbreaks) below and exceeding 20 per 100,000 cases per week, respectively.

## Results

Through direct communication with pulmonary diagnostic laboratories across Canada, we found wide variation in the delivery of these services during the pandemic and in the post-peak phase. We noted variable practices in screening procedures, COVID-19 testing, and PPE (procedural/surgical masks vs N95 respirators) across laboratories within and between institutions. We searched for international,^2–4^ national,^1-5^ and provincial^6^ guidelines that provided recommendations for low or moderate-to-high community prevalence settings. Only 1 guideline provided recommendations for both settings.^4^ All were opinions from respiratory expert bodies and regarded these procedures as high risk for airborne infection transmission. As such, enhanced PPE and airborne precautions were recommended. No direct data were available regarding the risk of SARS-CoV-2 transmission related to pulmonary diagnostic procedures. Table 1 highlights the recommendations that were developed through consensus with stakeholders and after consideration of the limited available evidence.

**Table 1.** Recommendations for Low and Moderate-to-High COVID-19 Prevalence Settings

Prescreening Questionnaire*	Prescreening questionnaire should be implemented at the time of informing patient of appointment, preferably 24–48 h prior to the test. Screening questionnaires will assess for any CHANGES or NEW symptoms as a potential indicator of active infection, or known COVID-19 exposures and as such will require COVID-19 testing Is the patient coming from an institution currently in outbreak or does the patient have a pending COVID-19 test because the patient is or was symptomatic? YES NO Did you/the patient have close contact with anyone with acute respiratory illness or travelled outside Ontario in the last 14 days? YES NO Have you/the patient have a confirmed case of COVID-19 or had close contact with a confirmed case of Covid-19? YES NO Do you/the patient have any of the following symptoms: Fever Sore throat Headache Chills Nausea/vomit, diarrhea, abdominal pain Unexplained fatigue, malaise, muscle aches (myalgias) New onset cough Worsening chronic cough Shortness of breath Difficulty breathing Difficulty swallowing Decrease or loss of sense of taste or smell Pink eye (conjunctivitis) Runny nose/nasal congestion without other known cause Red/purple lesions on hands/feet NO YES—if yes, please circle the symptom(s) If the patient is 70 years of age or older, are they experiencing any of the following symptoms: delirium, unexplained or increased number of falls, acute functional decline, or worsening chronic conditions? NO YES			
Patient risk stratification		High COVID-19 risk	Low COVID-19 risk	Negligible COVID-19 risk
	NPS result OR symptoms OR exposure history	NPS positive	NPS pending	NPS negative and no exposures
		One of the following: Symptoms not explained by other diagnosis Unable to get a history COVID-19 exposed and any symptoms	One of the following: Symptoms explained by another diagnosis COVID-19 exposed and NO symptoms	One of the following: No exposures AND no symptoms (no NPS unless AGMP planned during moderate-to-high prevalence) Resolved case of COVID-19 >14 d but <3 months ago (immune)
	Action	Postpone for minimum 14 d AND improved symptoms for 72 h	Postpone testing until NPS result available	Proceed with testing
Testing modality		Exercise stress testing	Cardiopulmonary exercise testing	PFT spirometry
Personal protective equipment (PPE)	Low prevalence	Droplets and contact precautions: surgical/procedural masks and face shield. Frequent hand washing, avoid touching of face/mucus membranes. Isolation gowns and gloves based on point of care risk assessment.		
	Mod-to-high prevalence					
Other considerations	• Plexiglass barriers or screen as a physical barrier may be considered where feasible • Cleaning instructions of flow sensors as per manufacturer’s instructions • High bacteria and pathogen filters for PFT spirometry equipment • Ensure room air exchange is 6–12 cycles/h^10^ • Modifications to procedural scheduling to accommodate for time needed for cleaning, ventilation, donning/doffing between PPE			
Cleaning procedures	Wiping of all surfaces with hospital grade disinfectants between patients within a 2-m radius of patients, or within 3 m if patients are in an elevated position, such as a treadmill. Settling time not required.			

Note. PFT, pulmonary function testing; NPS, nasopharyngeal swabs for PCR detection of SARS-CoV-2.

## Discussion

As cases of COVID-19 fluctuate, it is important to provide infection-specific guidance to facilitate the care of patients with cardiopulmonary disease. In our literature search, we found robust evidence supporting the generation of respiratory aerosols and droplets of varying sizes with coughing, sneezing, speech, singing, and breathing.^7^ There were no large or robust studies for the level of aerosols and respiratory droplets generated with spirometry or exercise testing. However, it is reasonable to assume that the deep exhalation and coughing associated with these pulmonary diagnostic procedures leads to aerosol and respiratory droplet generation. All previous guidelines have cautioned against these procedures as high-risk for airborne transmission and have recommended N95 respirators for protection. This recommendation is further supported by detectable SARS-CoV-2 viral RNA and viable virus within aerosol-droplet surrounding areas distant from infected patients, suggesting the potential for airborne transmission.^8^ However, no data to date have indicated that droplets carry sufficient viable virus to result in infection transmission; thus, it is not relevant in the healthcare setting. Furthermore, no strong data support meaningful reduction in transmission risk with use of N95 respirator instead of surgical masks for non-AGPs. For these reasons, we recommend the use of droplet and contact precautions with surgical or procedural masks and eye protection for these diagnostic procedures. If the point-of-care risk assessment suggests potentially elevated exposure risk, then isolation gowns and gloves may be added in a low-prevalence setting. In settings with low and moderate-to-high prevalence, pulmonary function testing should be deferred in patients who are risk-stratified as high risk for COVID-19.

Similar to other guidelines, we recommend that all facilities ensure that current recommended standards for heating, ventilation, and air conditioning are met, including temperature, humidity, and air changes. Environmental conditions likely play a role in cases in which aerosol transmission is the predominant mode of COVID-19 infection.^9^ Furthermore, under laboratory conditions, these environmental factors have been shown to determine the travel trajectory of expired particles.^10^ Lastly, universal masking and physical distancing should be maintained.

In conclusion, we developed COVID-19–specific recommendations for restoring exercise and PFT using a process of consensus involving all relevant key stakeholders becase there are no data available to inform the risk of SARS-CoV-2 transmission related to these pulmonary diagnostic procedures. Our guidance document incorporates a patient- and community-level risk-stratified approach that will facilitate the uniform adoption of IPAC practices across laboratories while protecting patients and staff in low- and high-prevalence settings.
